# Nutrient patterns are associated with discordant apoB and LDL: a population-based analysis

**DOI:** 10.1017/S000711452100369X

**Published:** 2022-08-28

**Authors:** Mohsen Mazidi, Richard J. Webb, Elena S. George, Niloofar Shekoohi, Julie A. Lovegrove, Ian G. Davies

**Affiliations:** 1 Department of Twin Research & Genetic Epidemiology, Kings College London, St Thomas, London, UK; 2 Medical Research Council Population Health Research Unit, University of Oxford, Oxford, UK. Clinical Trial Service Unit and Epidemiological Studies Unit (CTSU), Nuffield Department of Population Health, University of Oxford, Oxford, UK; 3 Faculty of Science, Liverpool Hope University, Liverpool, L16 9JD, UK; 4 Institute for Physical Activity and Nutrition, School of Exercise and Nutrition Sciences, Deakin University, Geelong, Australia; 5 Department of Cellular and Molecular Nutrition, School of Nutritional Sciences and Dietetics, Tehran University of Medical Sciences, Tehran, Iran; 6 Hugh Sinclair Unit of Human Nutrition and Institute for Cardiovascular and Metabolic Research, Harry Nursten Building, University of Reading, Pepper Lane Reading, UK; 7 School of Sports and Exercise Sciences, Faculty of Science, Liverpool John Moores University, Liverpool, UK

**Keywords:** apoB, LDL-cholesterol, Lipoprotein, Nutrition, Diet, Discordance, Nutrient pattern, NHANES

## Abstract

Individuals with discordantly high apoB to LDL-cholesterol levels carry a higher risk of atherosclerotic CVD compared with those with average or discordantly low apoB to LDL-cholesterol. We aimed to determine associations between apoB and LDL-cholesterol discordance in relation to nutrient patterns (NP) using National Health and Nutrition Examination Survey data. Participants were grouped by established LDL-cholesterol and apoB cut-offs (Group 1: low apoB/low LDL-cholesterol, Group 2: low apoB/high LDL-cholesterol, Group 3: high apoB/low LDL-cholesterol, Group 4: high apoB/high LDL-cholesterol). Principle component analysis was used to define NP. Machine learning (ML) and structural equation models were applied to assess associations of nutrient intake with apoB/LDL-cholesterol discordance using the combined effects of apoB and LDL-cholesterol. Three NP explained 63·2 % of variance in nutrient consumption. These consisted of NP1 rich in SFA, carbohydrate and vitamins, NP2 high in fibre, minerals, vitamins and PUFA and NP3 rich in dietary cholesterol, protein and Na. The discordantly high apoB to LDL-cholesterol group had the highest consumption of the NP1 and the lowest consumption of the NP2. ML showed nutrients that had the greatest unfavourable dietary contribution to individuals with discordantly high apoB to LDL-cholesterol were total fat, SFA and thiamine and the greatest favourable contributions were MUFA, folate, fibre and Se. Individuals with discordantly high apoB in relation to LDL-cholesterol had greater adherence to NP1, whereas those with lower levels of apoB, irrespective of LDL-cholesterol, were more likely to consume NP3.

A high concentration of fasted serum LDL-cholesterol is an independent causal risk factor for atherosclerotic CVD (ASCVD)^(1)^. A reduction in this, with a concordant reduction in LDL particle number (or apoB concentration) has been shown to reduce the risk of ASCVD^(1)^. However, a considerable proportion of patients with ASCVD show normal/low LDL-cholesterol levels and, despite achieving significant LDL-cholesterol reduction with lipid-lowering agents, many still encounter ASCVD events (residual risk)^(2,3)^. A phenomenon that may be associated with the presence of an increased number of circulating small dense LDL particles, which are more atherogenic^(4)^. Conversely, others may have higher LDL-cholesterol and lower apoB, a profile indicative of a smaller number of LDL particles that are larger in size, which result in a reduced ASCVD risk^(3)^. In this regard, it has been shown that the size of atherogenic lipoprotein particles is more discriminatory, as smaller particles more readily penetrate the endothelial wall and have greater susceptibility to atherogenic modification than their larger counterparts; a process that initiates and drives ASCVD^(5)^. Therefore, LDL-cholesterol may not always be a reliable predictor of ASCVD.

ApoB is a component of all atherogenic particles and has been proposed as an appropriate surrogate for particle number, due to the presence of one apoB molecule per lipoprotein particle^(6)^. Epidemiologic studies show that the rate of plaque progression and occurrence of ASCVD events is associated with the level and duration of exposure to apoB lipoproteins^(7,8)^. Furthermore, the application of Mendelian randomisation has shown apoB to be the predominant trait responsible for the relationship between lipoproteins and the progression of ASCVD^(9)^. Moreover, the risk of ASCVD is more strongly associated with the number of apoB particles rather than their cholesterol content^(10–14)^. This risk is also increased by the presence of additional molecules bound to the apoB-containing lipoprotein complex, for example, oxidised phospholipids, which have been shown to have major proatherogenic and proinflammatory roles^(15)^. Therefore, apoB is arguably a superior risk marker for ASCVD than LDL-cholesterol^(16)^. Furthermore, it is important to note that a Western dietary pattern, typically rich in SFA and refined carbohydrates, has also been shown to further intensify this atherogenic milieu, as well as exacerbating other associated risk factors, such as dysregulating glucose homoeostasis and unfavourable body composition^(17)^.

The application of machine learning (ML) for the evaluation of nutrient patterns (NP) offers advantages compared with traditional techniques, including the identification of novel trends and patterns derived from models built and operated without human intervention or assistance, unlike traditional statistical approaches where human intervention is essential at every stage of the model-building process^(18)^. ML techniques also offer superior handling of multi-dimensional and large data^(18)^. Indeed, ML is a valuable tool for the evaluation of disease risk in nutritional epidemiology as it offers enhanced prediction of clinically meaningful risk factors and the identification of predictive patterns related to diet^(18)^. Structural equation models (SEM) offer an improved ability to determine complex networks (magnitude of associations), as well as benefitting from multicollinearity and other features not possible with traditional statistical models^(19)^.

There is a lack of evidence regarding the dietary intake of individuals stratified according to the extent of concordance/discordance of apoB and LDL-cholesterol concentrations. Research addressing this would therefore advance dietary recommendations towards precision/personalised nutrition, especially for individuals with discordantly high levels of apoB in relation to LDL-cholesterol. Accordingly, in this population-based study, we aimed to determine, for the first time, the associations between LDL-cholesterol and apoB discordance in relation to NP by employing principle component analysis and a ML approach in conjunction with SEM. We hypothesised that discordantly high apoB compared with LDL-cholesterol will be associated with a poorer overall NP.

## Methods

### Population

The National Health and Nutrition Examination Survey (NHANES) is an ongoing, repeated set of cross-sectional surveys conducted by the National Center for Health Statistics (NCHS). NHANES uses a multistage probabilistic sampling strategy that oversamples certain segments of the population, including African-Americans, Mexican-Americans and those of lower socio-economic status. Approximately 5000 subjects are recruited into NHANES each year, and the data are publicly available in 2-year cycles. The present study used data pertaining to two 2-year NHANES survey cycles between 2005 and 2012, restricted to participants aged ≥ 18 years. Demographic, dietary and behavioural information are gathered through in-home questionnaires, while anthropometric and biomarker data are collected by trained staff using mobile examination units. The NCHS Research Ethics Review Board approved the underlying protocol, and written informed consent was obtained from all subjects. The interview consists of questions on socio-demographic characteristics and previously diagnosed medical conditions. More detailed information on the NHANES survey design and questionnaires is reported elsewhere^(20)^. A blood sample was drawn from the participants’ antecubital vein. Details of anthropometry procedures and laboratory procedures for collection, storage, calibration and quality control of blood samples are available elsewhere^(20)^. Blood samples are analysed for a large number of markers in NHANES; however, only those pertaining to glucose, insulin and lipid parameters were used in the present analysis.

### Dietary assessment

Dietary intake was assessed using 24-h recall and was obtained by a trained interviewer during the mobile examination centre visit with the use of a computer-assisted dietary interview system with standardised probes, i.e. the USDA’s automated multiple-pass method, as described previously^(21)^. Briefly, the type and quantity of all foods and beverages consumed in a single 24-h period before the dietary interview (from midnight to midnight) at the mobile examination centre were collected with the use of automated multiple-pass method. Automated multiple-pass method is designed to enhance complete and accurate data collection while reducing the respondent burden^(21,22)^.

### Statistical analysis

Participants were stratified by established LDL-cholesterol and apoB cut-offs (130 mg/dl and 160 mg/dl for apoB and LDL-cholesterol, respectively) into four discordant groups (Group 1: low apoB, low LDL-cholesterol; Group 2: low apoB, high LDL-cholesterol; Group 3: high apoB, low LDL-cholesterol; Group 4: high apoB, high LDL-cholesterol)^(23)^. We then conducted further analyses according to the guidelines published by the Centres for Disease Control for analysis of complex NHANES data set accounting for the masked variance and using the proposed weighting methodology^(24)^. The energy intake of the whole group of participants was adjusted using the residuals method, and any individuals with an energy intake above or below 2 SD of the mean energy intake were excluded from the analysis. Factor analysis with orthogonal transformation (varimax procedure) was applied. This was used to derive NP based upon the nutrients consumed by participants, as opposed to food items which are often used in dietary pattern analysis. We used factor analysis with Varimax orthogonal transformation to generate principle components representative of NP based on the highest correlation coefficients between the nutrients constructing each principle component^(25)^. All the necessary prerequisites of principle component analysis including linearity, Kaiser–Meyer–Olkin measure of 0·88 and the significant Bartlett’s test of sphericity (*P* < 0·001) were met. We then used regression methods to calculate the factor scores of each NP for each study participant^(25)^. Higher score means greater adherence to particular NP. Factors were retained for further analysis based on their natural interpretation and eigenvalues on the Scree test^(26,27)^. We computed the factor score for each NP by summing up intakes of nutrients weighted by their factor loadings^(26,27)^. Each participant received a factor score for each identified pattern^(28)^. A cut-off value for absolute factor scores of ≤ 0·25 was adhered to because it has been demonstrated as the optimum value for ensuring the best model fit^(29)^ and has been successfully used previously^(30,31)^. Continuous and categorical demographic variables were compared across apoB and LDL-cholesterol discordant groups using ANOVA and *χ*^2^ tests, respectively. We computed adjusted mean intakes of NP and nutrients using ANCOVA with a Bonferroni correction with two different levels of adjustment (model 1: adjusted for age, sex and ethnicity; model 2: adjusted for age, sex, ethnicity, poverty:income ratio, alcohol intake, smoking, BMI, physical activity, fasting blood glucose, systolic and diastolic blood pressure, hypertension (diagnosed in individuals with systolic blood pressure ≥ 140 mmHg, a diastolic blood pressure ≥ 90 mmHg or in those on antihypertensive drugs) and diabetes mellitus (self-reported history of DM or fasting plasma glucose ≥ 126 mg/dl)). ANCOVA allowed us to estimate the mean of our interested variable whilst adjusting for potential covariates.

### Machine learning

We used ML to assess the most important dietary variables for our outcomes; a composite score of LDL-cholesterol and apoB discordance, which is referred to as ‘joined effect’. We hypothesised that each dietary variable may have a different effect on the joined effect of apoB and LDL-cholesterol. Therefore, we implemented our model for each of the four groups separately to reveal predictors of the outcomes (the joined effect of apoB and LDL-cholesterol was calculated using the dimension reduction method, principal component analysis). A random forest (RF) model was applied with cross validation. This method fits many classification trees to a data set, then combines the predictions from all trees to present a final predictive model that ranks variables by their predictive power. For the evaluation of our models we have used *R*^2^ and *Q*^2^ (an estimate of the predictive ability of the model calculated by cross-validation). A negative *Q*^2^ means the model is not at all predictive. Model performance was confirmed by permutation analysis (*n* 1000).

### Structural equation modeling

We used structural equation modeling (sem) to test the overall model fit and relationships between sets of variables which were selected from machine learning to understand the underlying relationship of the composite score of apoB and LDL-cholesterol (for each group separately). SEM are able to test the fit of the defined model based on the observed covariance between the variables. We fitted our model under a maximum likelihood framework using covariance matrices^(32)^. All continuous variables were standardised by rank-normal transformed (mean 0, sd 1) by age, energy and sex (and by medication history). Relative model fit was assessed using the comparative fit index (CFI); a model with a ‘good’ fit typically requires indices to exceed 0·95. Absolute fit was assessed using the root mean square error of approximation (RMSEA). This ranges from 0 to 1, with 0 indicating a perfect fit^(32)^. A poorly fitting model is typically defined by RMSEA > 0·06^(33)^. CFI and RMSEA were not used to formally determine adequacy of fit, as their use in this context is controversial and there is limited consensus on appropriate cut-off values because each index is affected differently by degrees of freedom, model complexity and sample size; however, it is standard practice to report these along with the *χ*^2^. Statistical analysis was performed in the R environment for statistical computing v 3.5.1 (R Foundation for Statistical Computing. https://www.R-project.org/). A two-sided *P* < 0·05 was used to characterise significant results.

## Results

### General characteristics

Participants were stratified by established LDL-cholesterol and apoB cut-offs into four discordant groups, as shown in Table 1^(23)^. Subjects in Group 3, high apoB and low LDL-cholesterol, had a significantly higher waist circumference (106·0 ± 2·00 cm), fasting blood glucose (117·7 ± 10·3 mg/dl), fasting blood insulin (16·8 ± 1·7 μU/ml) and HOMA-IR (5·2 ± 0·9) compared with Group 4, high apoB and high LDL-cholesterol (all *P* < 0·001).

**Table 1.** Demographic and clinical characteristics of the total population grouped by apo B and LDL-cholesterol levels (Mean values and standard errors of the mean; percentages)

Characteristics* †	Group 1 (low apoB, low LDL-cholesterol) (*n* 12 384)		Group 2 (low apoB, high LDL-cholesterol) (*n* 711)		Group 3 (high apoB, low LDL-cholesterol) (*n* 285)		Group 4 (high apoB, high LDL-cholesterol) (*n* 885)		*P*-value
	Mean	sem	Mean	sem	Mean	sem	Mean	sem	
Age (years)	48·8	1·2	51·1	1·9	55·6	1·4	56·1	1·6	< 0·001
Sex									
Male									
%	49·4		52·3		51·4		44·9		< 0·001
Female									
%	50·6		47·7		49·6		55·1		
Anthropometric parameters									
BMI (kg/m^2^)	28·3	0·1	28·1	0·5	30·9	0·9	30·1	0·6	< 0·001
WC (cm)	97·3	0·3	96·7	1·2	106·0	2·0	102·6	1·2	< 0·001
Insulin and glucose parameters									
Fasting blood glucose (mg/dl)	101·0	0·7	99·2	2·3	117·7	10·3	112·5	4·3	< 0·001
Plasma insulin (μU/ml)	13·1	0·3	11·2	0·6	16·8	1·7	13·1	0·9	< 0·001
HOMA-IR	3·4	0·1	2·8	0·2	5·2	0·9	3·6	0·3	< 0·001
HbA1c (%)	5·7	0·0	5·7	0·1	6·2	0·3	6·2	0·1	< 0·001
Macronutrients									
Fat (g/d)	78·5	1·0	75·5	4·2	78·6	6·4	69·2	3·3	< 0·001
MUFA (g/d)	29·2	0·4	28·7	1·7	31·3	3·0	25·7	1·2	< 0·001
PUFA (g/d)	17·1	0·2	15·3	1·0	15·7	1·5	15·2	0·8	< 0·001
SFA (g/d)	25·4	0·4	24·7	1·4	24·7	1·9	22·4	1·2	< 0·001
Protein (g/d)	79·2	0·9	77·8	3·7	76·1	5·8	70·4	3·2	< 0·001
Carbohydrate (g/d)	251·5	2·7	237·5	10·9	256·7	13·6	223·3	8·8	< 0·001
Fibre (g/d)	15·7	0·2	12·6	0·6	14·5	1·4	14·0	0·8	< 0·001
Total sugar (g/d)	113·5	1·7	112·3	7·1	126·1	10·3	103·2	5·5	<0·001
Energy (kcal/d)	2069·6	21·5	1992·4	87·9	2048·2	116·1	1823·1	69·3	< 0·001
Total cholesterol (mg/dl)	185·1	0·7	250·8	1·9	239·5	3·0	281·3	2·6	< 0·001
HDL-cholesterol (mg/dl)	54·1	0·3	56·3	1·3	42·9	1·8	51·4	1·1	<0·001
LDL-cholesterol (mg/dl)	105·4	0·6	170·4	0·9	145·0	2·9	192·1	2·4	< 0·001
apoB (mg/dl)	87·0	0·9	118·6	0·7	137·9	0·9	145·6	0·6	< 0·001
TAG (mg/dl)	116·0	1·3	117·0	4·7	232·9	12·9	176·6	5·3	< 0·001
TAG:HDL ratio	2·5	0·0	2·3	0·1	5·9	0·5	3·7	0·2	< 0·001
LDL-cholesterol/apoB ratio	1·2	0·0	1·5	0·0	1·1	0·0	1·3	0·0	< 0·001
Non-HDL-C	131·0	0·7	194·5	1·4	196·5	2·9	229·9	2·5	< 0·001

* Abbreviations: WC, waist circumference, HOMA-IR, Homeostatic Model Assessment of Insulin Resistance, HbA1c, glycated Hb.

† Continuous and categorical demographic variables were compared across the four groups using ANOVA and *χ*^2^ tests, respectively.

### Generation of principle components

Using the principle component method, we reduced the dietary intake from twenty-nine macronutrients and micronutrients into three NP that together explained 63·2 % of the variance of NP consumption. Loading factors and scree plot are shown in Supplementary Table 1 and Supplementary Fig. 1. The first NP (NP1) was characterised by being high in SFA, carbohydrate and low in most vitamins, the second pattern (NP2) contained high amounts of fibre, minerals, vitamins, MUFA and PUFA and the third NP was high in cholesterol, protein and Na (NP3).

As shown in Table 2, there was a significant difference between the adjusted mean score of the NP1 and NP2 (both *P =* <0·001). In the model adjusted for age, sex and ethnicity, regarding NP1 and NP2, Group 3 (high apoB, low LDL-cholesterol) consumed diets containing predominantly SFA and carbohydrate (mean score = 2965 ± 22) and cholesterol, protein and Na (mean score = 129 ± 20). Conversely, Group 1 (low apoB, low LDL-cholesterol) consumed the lowest amounts of the NP1 (mean score = 2237 ± 27) and NP3 (mean score = 115 ± 10). However, NP2, which was high in fibre, minerals, vitamins and PUFA, was consumed predominantly by Group 1 (mean score = 3626 ± 40) and Group 2 (mean score = 3656 ± 40). Whereas Group 3 was shown to consume this NP the least (mean score = 2969 ± 31), as shown in Table 2. Similar findings were observed with the fully adjusted model 2, as shown in Table 2.

**Table 2.** Adjusted mean of score of nutrient patterns grouped by apo B and LDL-cholesterol concentrations (Mean values and standard errors of the mean; percentages)

Variables	Group 1 (apoB: L, LDL-cholesterol: L)				Group 2 (apoB: L, LDL-cholesterol: H)				Group 3 (apoB: H, LDL-cholesterol: L)				Group 4 (apoB: H, LDL-cholesterol: H)				*P*-value
	Model 1*		Model 2†		Model 1*		Model 2†		Model 1*		Model 2†		Model 1*		Model 2†		
	Mean	SE	Mean	SE	Mean	SE	Mean	SE	Mean	SE	Mean	SE	Mean	SE	Mean	SE	
Nutrient pattern 1 (High in saturated fatty acids and carbohydrate)‡	2237	27	2196	22	2336	30	2289	26	2965	22	2896	20	2746	30	2654	27	Both <0·001
Nutrient pattern 2 (High in fibre, minerals vitamins, PUFA)‡	3626	40	3562	34	3656	40	3596	36	2969	31	2744	30	3125	30	2965	26	Both <0·001
Nutrient pattern 3 (High in cholesterol, protein, sodium)	115	10	99	9	124	10	114	10	129	20	118	19	119	20	103	17	Model 1 = 0.183 Model 2 = 0.249

* Model 1: Age, sex and ethnicity.

† Model 2: model 1 plus poverty:income ratio, alcohol intake, smoking, BMI, physical activity, fasting blood glucose, systolic and diastolic blood pressure, hypertension and diabetes mellitus.

‡ Dietary patterns were calculated using principle component analysis and variables were compared across the groups using ANCOVA test.

### Machine learning and structural equation model approach

We used ML to assess which dietary variables (online Supplementary Table 1) influenced apoB and LDL-cholesterol discordance using the joined effect of apoB and LDL-cholesterol (composite score) as a response for each discordant group separately. Since we hypothesise that each independent factor might have a varied effect on the level of apoB and LDL-cholesterol, we implemented our model for each of the four groups separately to reveal the predictors of LDL discordance based on apoB and LDL-cholesterol.

The most important variables for Group 1 were PUFA, MUFA, total carbohydrate, Cu, vitamin C and riboflavin (model performance: 0·86) and for Group 2 the variables of main importance were total sugars, total fat, fibre, SFA, cholesterol, vitamin A and vitamin E (model performance: 0·69). For Group 3, the most prominent variables were MUFA, total fat, SFA, fibre, Se, folate vitamin B_12_ and thiamine (model performance: 0·71), whereas for Group 4 they were total carbohydrate, protein, cholesterol, vitamin C, vitamin K, P and Mg (model performance: 0·56). Furthermore, we also performed this analysis on the whole population, which revealed that total sugars, SFA, fibre, total fat, cholesterol, vitamin A, vitamin C and Mg were the most important variables (model performance: 0·62).

To understand the magnitude of each nutrient with our outcome (joined effect of apoB and LDL-cholesterol), SEM were implemented for each group separately, as shown in Table 3. Regarding Group 1 (low apoB, low LDL-cholesterol), our findings showed that PUFA (*β* = –0·174, *P* < 0·001), MUFA (*β* = –0·104, *P* < 0·001) and total carbohydrate (*β* = 0·116, *P* < 0·001) were the only variables which had an interdependent significant association with our outcome (*χ*^2^ : 22·0, CFI: 0·97, RMSEA: 0·07). Total sugars (*β* = 0·172, *P* < 0·001), total fat (*β* = 0·019, *P* < 0·001), SFA (*β* = 0·400, *P* < 0·001), vitamin A (*β* = 0·137, *P* = 0·012) and fibre intake (*β* = –0·585, *P* < 0·001) had a significant independent link with our outcome in Group 2 (low apoB, high LDL-cholesterol) (*χ*^2^: 20·1, CFI: 0·98, RMSEA: 0·06). SEM revealed that MUFA (*β* = –0·766, *P* < 0·001), total fat (*β* = 0·721, *P* < 0·001), SFA intake (*β* = 0·242, *P* < 0·001), fibre (*β* = –0·411, *P* < 0·001), folate (*β* = –0·198, *P* = 0·031), Se (*β* = –0·133, *P* = 0·046) and thiamine (*β* = 0·146, *P* = 0·041) had a significant association with our outcome in Group 3 (high apoB, low LDL-cholesterol) (*χ*^2^: 12·3, CFI: 0·98, RMSEA: 0·04). In Group 4 (high ApoB, high LDL-cholesterol), SEM revealed that total carbohydrate (*β* = 0·442, *P* < 0·001), protein (*β* = 0·296, *P* < 0·001) and cholesterol (*β* = 0·294, *P* < 0·001) had a significant association with our outcome (*χ*^2^: 20·2, CFI: 0·97, RMSEA: 0·05). On the scale of the whole population, total sugars (*β* = 0·296, *P* < 0·001), SFA (*β* = 0·282, *P* < 0·001), fibre (*β* = –0·423, *P* < 0·001), cholesterol (*β* = 0·139, *P* < 0·001) and Mg (*β* = 0·114, *P* < 0·001) were significantly associated with our outcome (*χ*^2^: 16·6, CFI: 0·95, RMSEA: 0·07).

**Table 3.** Effect estimates of associations between nutrients and the joined effect of apoB and LDL-cholesterol

Exposure	Outcome	*β* coefficient	se	*P*-value
Group 1 (Low apoB, Low LDL-cholesterol)*				
Copper	apoB/LDL-cholesterol	0·022	0·509	0·136
MUFA	apoB/LDL-cholesterol	–0·104	0·022	< 0·001
PUFA	apoB/LDL-cholesterol	–0·174	0·034	< 0·001
Riboflavin	apoB/LDL-cholesterol	0·050	0·314	0·425
Total carbohydrate	apoB/LDL-cholesterol	0·116	0·003	< 0·001
Vitamin C	apoB/LDL-cholesterol	–0·017	0·004	0·235
Group 2 (Low apoB, High LDL-cholesterol)*				
Cholesterol	apoB/LDL-cholesterol	0·038	0·004	0·253
Fibre	apoB/LDL-cholesterol	–0·585	0·128	< 0·001
SFA	apoB/LDL-cholesterol	0·400	0·054	< 0·001
Total fat	apoB/LDL-cholesterol	0·264	0·019	< 0·001
Total sugars	apoB/LDL-cholesterol	0·172	0·011	< 0·001
Vitamin A	apoB/LDL-cholesterol	0·137	0·002	0·012
Vitamin E	apoB/LDL-cholesterol	0·103	0·203	0·098
Group 3 (High apoB, Low LDL-cholesterol)*				
Fibre	apoB/LDL-cholesterol	–0·411	0·099	< 0·001
Folate	apoB/LDL-cholesterol	–0·198	0·006	0·031
MUFA	apoB/LDL-cholesterol	–0·766	0·046	< 0·001
Se	apoB/LDL-cholesterol	–0·133	0·010	0·046
SFA	apoB/LDL-cholesterol	0·242	0·072	< 0·001
Thiamine	apoB/LDL-cholesterol	0·146	0·056	0·041
Total fat	apoB/LDL-cholesterol	0·721	0·022	< 0·001
Group 4 (High apoB, High LDL-cholesterol)*				
Cholesterol	apoB/LDL-cholesterol	0·294	0·004	< 0·001
Mg	apoB/LDL-cholesterol	–0·189	0·007	0·074
P	apoB/LDL-cholesterol	0·142	0·002	0·163
Protein	apoB/LDL-cholesterol	0·296	0·027	< 0·001
Total carbohydrate	apoB/LDL-cholesterol	0·442	0·010	< 0·001
Vitamin C	apoB/LDL-cholesterol	0·023	0·012	0·295

* To determine effects structural equation models were implemented for each group separately based upon the joined effect of apoB and LDL-cholesterol.

## Discussion

The impact of diet upon apoB and LDL-cholesterol discordance is currently unknown. Therefore, the aim of the present study was to apply two methods of determining NP to data derived from NHANES to reveal, for the first time, the association between our outcome (i.e. the joined effect of apoB and LDL-cholesterol) and nutrient intake between four groups stratified by apoB and LDL-cholesterol. Even after accounting for a large range of confounding variables, individuals with high apoB, irrespective of LDL-cholesterol, had the highest consumption of NP1, which was high in SFA and carbohydrates and low in vitamins and minerals and is characteristic of a ‘Western dietary pattern’. Furthermore, those with discordantly high levels of apoB in relation to LDL-cholesterol had the greatest consumption of NP3 (i.e. high in dietary cholesterol, protein and Na) and the lowest adherence to a healthier dietary pattern rich in fibre, minerals vitamins, MUFA and PUFA (i.e. NP2), which has characteristics of a Mediterranean-style diet.

In addition to using a traditional statistical approach, (i.e. PCA), for extracting patterns, we also implemented a novel ML approach in conjunction with SEM. The advantages of employing ML is that it can better explain intra-relationships and enable more accurate predictions to be made based upon the classification ability of the technique. SEM were then used to elucidate the magnitude of the resulting relationships, allowing us to determine nutrient predicators relating to the concordance/discordance of apoB and LDL-cholesterol. Our findings revealed that the groups with increased apoB concentrations were positively associated with a range of nutrients, including dietary carbohydrate, SFA, protein and cholesterol and negatively associated with other nutrients, such as MUFA and fibre; a finding also reflected in our analysis across the spectrum of the whole population. In agreement with these results, a previous analysis of dietary patterns in 140 healthy men demonstrated that switching dietary intake from a moderate carbohydrate/high fat diet to a high carbohydrate/low fat diet was associated with an increase in plasma apoB lipoprotein concentration^(34)^. The effect of these dietary changes on plasma apoB concentration was reported to be mediated via an increased flux of carbohydrate to the liver, which led to upregulated de-novo lipogenesis and the subsequent production of apoB lipoproteins^(35)^. Moreover, refined carbohydrate in particular has also been shown to result in increased visceral adiposity, decreased insulin sensitivity and the upregulation of hepatic de novo lipogenesis^(36)^. This consequently decreases HDL-cholesterol levels and raises apoB and LDL-cholesterol; in particular the small, dense LDL subclass, a particularly atherogenic lipoprotein, which is considered a key risk factor for ASCVD^(36)^. This constellation of lipid abnormalities, which also include raised circulating triacylglycerol concentrations are often referred to as atherogenic dyslipidaemia and is the result of neutral lipid exchange^(37)^. Therefore, the combination of a high intake of refined carbohydrates, free sugars and dietary fats such as SFA and trans fats, can be regarded as dietary components principally implicated in the the development of atherosclerosis. The presence of some of these elements in the nutrient pattern predominantly consumed by those with raised apoB, although somewhat unsurprising, should still be acknowledged.

Similarly, our ML analysis, followed by SEM, revealed a strong, positive and significant association between those with high apoB, low LDL-cholesterol and total dietary fat, whereas MUFA had a strong and significant negative association. A significant, yet weak positive relationship regarding SFA was also reported within this group. These findings are in contrast to previous studies which have observed different associations between saturated and unsaturated fatty acids and apoB^(38)^. For example, it has been reported that a diet high in SFA compared with a diet rich in *n*-6 PUFA contributed towards higher plasma proprotein convertase subtilisin/kexin type 9 (PCSK9) concentrations^(39)^, leading to a decrease in the catabolic rate of apoB^(40)^. It has also been suggested that unsaturated trans fatty acids, such as elaidic acid and conjugated linoleic acid, may increase the hepatic secretion of apoB-containing lipoproteins, mainly consisting of the small, dense LDL subclass^(41,42)^. Despite these findings, the weak positive relationship found between SFA and our outcome (i.e. the joined effect of apoB and LDL-cholesterol) in our study may be an indicator that the impact of SFA may be less than that inferred by unsaturated fatty acids upon atherogenic lipoproteins. Incidentally, a Mediterranean-style dietary pattern which typically contains lower levels of SFA, together with higher levels of MUFA and PUFA, has been associated with a beneficial increased clearance of apoB particles^(40)^, facilitated by a higher LDL receptor activity. Furthermore, this dietary pattern is effective for reducing endpoint ASCVD^(43–45)^.

With respect to those with low apoB, low LDL-cholesterol (Group 1), the highest association observed was consumption of NP2, which contains elements often found in a ‘Mediterranean-style diet’ (e.g. higher fibre, minerals, vitamins and PUFA). It is noteworthy that Group 2 (low apoB, high LDL-cholesterol) was also associated with consumption of this NP, despite having raised LDL-cholesterol. This is important since the benefits of a Mediterranean-style diet (which contains these key nutrients), and the positive effect on apoB has been well established^(38)^. For example, randomised controlled trials have shown that adherence to a Mediterranean diet for 3 months resulted in significant reductions in apoB^(46,47)^. Similarly, two prospective intervention studies demonstrated the favourable impact of the Mediterranean diet upon plasma apoB^(40,48)^. These improvements in apoB are likely to be the result of an increased consumption of pulses, vegetables and fruits, fish, contributing to an increase in unsaturated fatty acids, soluble fibre and polyphenols, as well as a decrease in SFA^(46)^. Moreover, favourable effects on apoB have been attributed to the diet being rich in extra virgin olive oil which, along with its phenolic compounds, has also been shown to influence mRNA and protein expression of lipoproteins, resulting in the reduced production of VLDL and their conversion to LDL lipoproteins, in tandem with a subsequent increase in the rate of LDL catabolism^(46,49)^. That said, it has previously been shown in the NHANES cohort the consumption of extra virgin olive oil is not high, and it is therefore questionable whether this nutritional aspect was responsible for the differences in apoB observed in this analyses^(50)^. Nonethless, regardless of the food items consumed a nutrient profile containing elements which are characteristic of a Mediterranean dietary pattern were found in the group with the lowest ASCVD profile in terms of apoB, which corresponds with the existing literature^(46)^.

Key strengths of our study include the large and nationally representative sample, which is adequately powered to determine associations^(51)^. Furthermore, the novel combination of ML and SEM approaches provide statistical metrics of magnitude and significance, which allow for inferences to be made regarding input variables and outcomes (i.e. joined effect of apoB and LDL-cholesterol)^(18)^. This is not the case when using ML methods alone, which often impedes the interpretation of findings^(18)^. In addition to this, our novel method for describing food intakes and nutrient patterns has also facilitated the elucidation of unique relationships and the quantification of novel NP, which would not otherwise have been possible. Despite these strengths, our study has some limitations. The dietary recall methods used can be prone to bias, particularly with regard to misreporting, which when present has been shown to affect all food groups^(52)^. Furthermore, associations do not imply causality and are acknowledged as a limitation of all observational studies and should be considered when evaluating our findings^(53)^. Despite best efforts, residual confounding from a range of lifestyle and socio-economic factors, along with bias, may also limit the generalisability of our findings^(54)^. Similarly, there is the possibility of reverse causaility in observational studies and even when taking precautions, such as excluding individuals with pre-existing illness, its influence cannot be entirely negated^(55)^. For example, it has been previously shown that the relationship between dietary and serum cholesterol can be confounded by reverse causality based upon education level and employment status^(56)^.

In conclusion, we have shown that those individuals with discordantly high levels of apoB in relation to LDL-cholesterol consume diets containing elements that may possibly represent a ‘Western’ dietary pattern. This includes high dietary SFA and refined carbohydrates and a low intake of fibre and minerals. These findings will be useful for personalised nutrition strategies in populations stratified by LDL-cholesterol and apoB concentrations. Furthermore, we have also demonstrated that those with lower levels of apoB were more likely to consume diets containing elements of a ‘Mediterranean-style’ dietary pattern, despite high LDL-cholesterol concentrations.

## Supplementary material

For supplementary material accompanying this paper visit http://doi.org/10.1017/S000711452100369X. click here to view supplementary material
