# Supplementary material for: Nutrient patterns are associated with discordant apoB and LDL: a population-based analysis
Source: Br J Nutr. 2021 Sep 15;128(4):712–20. doi: 10.1017/S000711452100369X (PMC9346615; doi:10.1017/S000711452100369X)
Supplement: Supplementary file 1 [file S000711452100369Xsup001.docx]

**Supplementary Data**

| **Supplementary Table 1: Factor-loading matrix for the 3 nutrient patterns identified among subjects** | | | |
| --- | --- | --- | --- |
| **Nutrients** | **First Nutrient Pattern** | **Second Nutrient Pattern** | **Third Nutrient Pattern** |
| **Protein** | ---- | ---- | 0.569 |
| **Carbohydrate** | 0.756 | ---- | --- |
| **Total sugars** | 0.695 | ---- | --- |
| **Dietary fiber** | --- | 0.912 | ---- |
| **Total fat** | 0.658 | 0.302 | 0.296 |
| **Saturated fatty acids** | 0.869 | ---- | --- |
| **Monounsaturated fatty acids** | ---- | 0.569 | --- |
| **Polyunsaturated fatty acids** | ----- | 0.869 | --- |
| **Cholesterol** | 0.436 | ---- | 0.639 |
| **Vitamin E** | 0.536 | 0.318 | --- |
| **Vitamin A** | 0.425 | 0.309 | --- |
| **Thiamin (Vitamin B1)** | --- | 0.326 | 0.298 |
| **Riboflavin (Vitamin B2)** | --- | 0.468 | 0.326 |
| **Niacin** | --- | 0.395 | 0.269 |
| **Vitamin B6** | --- | 0.296 | --- |
| **Total folate** | --- | 0.469 | --- |
| **Vitamin B12** | --- | 0.536 | 0.342 |
| **Vitamin C** | ---- | 0.429 | --- |
| **Vitamin K** | 0.369 | 0.369 | --- |
| **Calcium** | ---- | 0.458 | --- |
| **Phosphorus** | ---- | 0.462 | --- |
| **Magnesium** | ----- | 0.526 | ---- |
| **Iron** | ----- | 0.365 | 0.326 |
| **Zinc** | ---- | 0.758 | --- |
| **Copper** | ----- | 0.625 | --- |
| **Sodium** | ---- | 0.326 | 0.469 |
| **Potassium** | ---- | 0.458 | --- |
| **Selenium** | ---- | 0.395 | --- |
| **Alcohol** | ---- | ----- | 0.369 |
| **Absolute values ≤0.25 were excluded from the table for simplicity.** | | | |


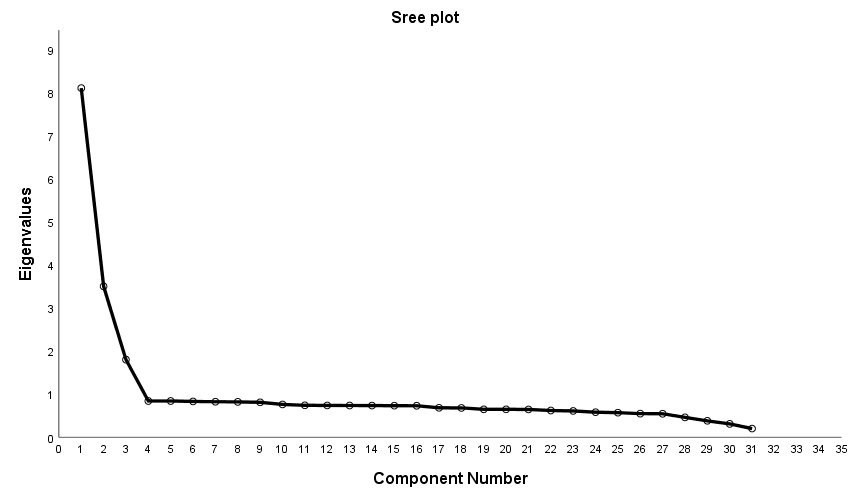


**Supplementary Figure 1.** Scree plot of eigenvalues resulting from principle component analysis.
